# Patients’ assessment of professionalism and communication skills of medical graduates

**DOI:** 10.1186/1472-6920-14-28

**Published:** 2014-02-11

**Authors:** Fatima T Abadel, Abdulla S Hattab

**Affiliations:** 1 Community medicine and public health department, Faculty of medicine and health sciences, University of Aden, Aden, Yemen

## Abstract

**Background:**

Professionalism and communication skills constitute important components of the integral formation of physicians which has repercussion on the quality of health care and medical education. The objective of this study was to assess medical graduates’ professionalism and communication skills from the patients’ perspective and to examine its association with patients’ socio-demographic variables.

**Methods:**

This is a hospital based cross-sectional study. It involved 315 patients and 105 medical graduates selected by convenient sampling method. A modified and validated version of the American Board of Internal Medicine’s (ABIM) Patient Assessment survey questionnaire was used for data collection through a face to face interview. Data processing and analysis were performed using the Statistical Package for Social Science (SPSS) 16.0. Mean, frequency distribution, and percentage of the variables were calculated. A non-parametric Kruskal Wallis test was applied to verify whether the patients’ assessment was influenced by variables such as age, gender, education, at a level of significance, p ≤ 0.05.

**Results:**

Female patients constituted 46% of the sample, whereas males constituted 54%. The mean age was 36 ± 16. Patients’ scoring of the graduate’s skills ranged from 3.29 to 3.83 with a mean of 3.64 on a five-point Likert scale. Items assessing the *“patient involvement in decision-making”* were assigned the minimum mean values, while items dealing with “*establishing adequate communication with patient*” assigned the maximum mean values. Patients, who were older than 45 years, gave higher scores than younger ones (p < 0.001). Patients with higher education reported much lower scores than those with lower education (p = 0.003). Patients’ gender did not show any statistically significant influence on the rating level.

**Conclusion:**

Generally patients rated the medical graduates’ professionalism and communication skills at a good level. Patients’ age and educational level were significantly associated with the rating level.

## Background

The importance of patients’ opinion in the evaluation of doctors’ professional performance and the quality of health care has been stressed in several studies [1-4]. The Accreditation Council for Graduate Medical Education (ACGME) has focused on the importance of residents’ competence in professionalism and interpersonal and communication skills [5,6]. Today, professionalism constitutes an indispensable quality for any practicing physician. Literature review emphasizes the following requirements for professionalism: integrity, honesty, compassion, a commitment to keeping current with medical advances, the ability to communicate effectively with patients, and to respect patient autonomy [7-12]. Communication with patients is the core clinical skill for the practice of medicine. It has been defined as specific tasks and observable behaviors that include interviewing to obtain a medical history, explaining a diagnosis and prognosis, giving therapeutic instructions and information needed for informed consent to undergo diagnostic and therapeutic procedures, and providing counseling to motivate participation in therapy or to relieve symptoms [13,14]. Respect for patients’ needs and wishes are central to any human health care system [15]. Clinical communication is complex in nature, and both personal and curricular factors will influence how medical students master the relevant skills [16]. Lakoff et al. argued that communication skills appear to be an integral part of one’s cognition where basic or general communication skills are developed early in life, but the theoretical knowledge about communication skills comes years later and not through medical studies alone [17].

Positive doctor-patient relationship can increase the patient’s perceptions of physician competence [18]. Research has shown that physicians who exhibit negative communication behaviors are more likely to have been sued in the past for malpractice than those with more positive doctor patient relationship [19-22]. Beckman, et al. found that in 70% of malpractice depositions, communication problems between physicians and patients were identified [23].

The assessment of patients’ perception of the medical graduates’ professionalism and communication skills during their first postgraduate years could provide an early indicator of the quality of the undergraduate curriculum and educational process and constitutes an important component of the evaluation of the quality of health care. These issues have been well documented in the medical literature [3,4,6,8,18,21,22]. To our knowledge, this is the first study of its kind to be performed in Yemen.

This study aimed at answering the following question: *How do patients assess medical graduates’ professionalism and communication skills in Aden hospitals?*

## Methods

### Study design

This is a cross-sectional hospital-based study conducted during the period from 1^st^ January to 30^th^ March, 2010. It constitutes a part of a larger study which deals with the different areas of medical graduates’ competency: professionalism, communication skills, clinical skills, population health, management of information, and critical thinking [24].

### Study setting

The study was carried out in eight hospitals’ outpatient clinics and inpatient wards, (four public and four private) in Aden city, where the medical graduates were working at the time of data collection.

Yemen is located at the south western corner of Asia. It has an area of 527,970 square kilometers, 23.85 million inhabitants, and is divided administratively into twenty one governorates. Aden governorate is the commercial and economic capital of the unified Yemen with nearly one million inhabitants. Geographically Aden is located at the south-western corner of the Arabian Peninsula. It overlooks the Arabian Sea to the south and the Red Sea to the west. Administratively, Aden governorate is divided into eight districts [25].

### The study population

This study was designed to include two different populations: medical graduates and patients.

#### Medical graduates

The study population covered all the medical graduates of Aden University during the period 2005–2009 who had studied the same curriculum and were working at Aden hospitals. One hundred and five graduates (61.9% females and 38.1% males) were included in the study. All of them were available at the time of data collection and gave their consent to participate in the study.

### Recruitment strategies

1. A list including the names of medical graduates (2005-2009) and their work location was obtained from hospitals’ administrations.

2. In coordination with the hospitals’ authorities and heads of the departments, meetings were organized with the potential research participants (medical graduates). In these meetings, a detailed explanation of the study objectives and methods were provided.

3. Potential research participants were asked to give their consent to have their performance (professionalism and communication skills) rated by some patients whom they cared for, without knowing which patients or when data will be collected. Accordingly verbal consent was obtained. None of the graduates who were approached declined participation in the study.

#### Patients

##### Sample size

The sample size was determined by convenient purposeful quota sampling method with which every graduate will be assessed by three different patients; accordingly the sample was 315 patients [26]. The participants were selected according to the following criteria: adult patient (18 years or older), fully conscious and verbally expressed willingness to participate in the study.

##### Sampling methods

A multi-stage stratified random sampling was performed. First, four districts were randomly selected. Then, two hospitals from each district were randomly selected (one public and one private hospital). Finally, the 315 patients were proportionally distributed according to the proportion of graduates in the selected hospitals. The sample frame for this study was the outpatients’ clinics and the inpatients’ wards of the selected hospitals where the medical graduates were working. The patients were selected by systematic random sampling method where every 3^rd^ patient was approached. If the patient refused to take part in the study or was ineligible, the next eligible patient was selected. Thirty nine patients declined participation in the study so they were substituted by others.

##### Data collection

Data were collected through a direct face to face interview. An exit interview was conducted with the hospitals’ outpatients immediately after leaving the consultation room. For the inpatients interviews were conducted at the moment of discharge. All interviews were conducted by the same researcher with the same method for all patients. The absence of time lag between consultations and interviews minimize or adjusts for recall bias. To guarantee the participants’ privacy, interviews were performed in a private side-room which was secured by a special arrangement with the hospitals’ administration.

##### Instrument

For data collection a validated Arabic version of the ABIM’s Patient Assessment survey questionnaire, which is a part of the Patient and Physician Peer Assessment Module for maintenance of certification, was used (Additional file 1) [27]. Different studies have validated the ABIM domains not only in the context in which it was originally developed in the USA [28-35] but also in other non-western countries Taiwan, Iran, Japan and Saudi Arabia [36-39].

Content validity of the survey questionnaire was examined by asking ten experts at the Faculty of Medicine to judge if the items cover all aspects of the domain intended to be measured. In addition, a pilot study was carried out and the internal consistency reliability of the questionnaire was calculated using (Cronbach’s alpha) and was found to be >0.9.

The questionnaire included ten questions about different aspects of professionalism and communication skills. Patients were asked to rate each of these aspects on a five-point Likert scale ranging from poor (being the lowest level of competency, scored 1) to excellent (being the highest level scored 5). The final version of the questionnaire appears in Table 1.

**Table 1:** Questionnaire for patients’ assessment of the different items of professionalism and communication skills of the medical graduates

**How was the physician’s performance at:**	**Excellent**	**V.G**	**Good**	**Fair**	**Poor**
1. Telling you every thing; being truthful, upfront and frank; not keeping things from you that you should know.					
2. Greeting you warmly; calling you by the name you prefer; being friendly, never crabby or rude.					
3. Treating you like you’re on the same level; never “talking down” to you or treating you like a child.					
4. Letting you tell your story ; listening carefully; asking thoughtful questions; not interrupting you while you’re talking.					
5. Showing interest in you as a person; not acting bored or ignoring what you have to say.					
6. Warning you during the physical exam about what he/she is going to do and why; telling you what he/ she finds.					
7. Discussing options with you; asking your opinion; offering choices and letting help decide what to do; asking what you think before telling you what to do.					
8. Encourage you to ask questions; answering them clearly; never avoiding your questions or lecturing you.					
9. Explaining what you need to know about your problems, how and why they occurred, and what to expect next.					
10. Using words you can understand when explaining any technical medical terms in plain language.					

### Ethical considerations

1. The study protocol was approved by “the Committee of Research and Postgraduate Studies, Faculty of Medicine and Health Science, Aden University” which is responsible for both ethical and scientific review *in compliance with Helsinki Declaration.*

2. Permission of the hospitals’ authorities where the study was conducted was obtained.

3. Verbal consent was obtained from all potential participants after providing them with detailed explanation of the objectives, importance and benefits of the research. They were also assured that all the collected data would be handled with full confidentiality. Furthermore, they were informed that they had the right to refuse participation, and/or to withdraw at any moment.

### Data analysis

Data processing was performed using the SPSS 16.0 software package. Multi items of competency for each participant were computed into singular mean and singular percentage. For the convenience of analysis, the five-point Likert scale was re-categorized into three groups: *inadequate, good* and *very good*, where the *inadequate group* combined the fair and poor scores and the *very good group* combined the very good and excellent scores.

The mean, frequency distribution and percentage of the variables were calculated. A non-parametric Kruskal Wallis test was applied to verify whether the raters’ assessments were influenced by variables such as patients’ age, sex, education and residency at a (p ≤ 0.05) level of significance.

## Results

A total number of 315 patients who attended the selected hospitals during the study period were interviewed; 54% of them were males. Female patients were younger than males. The mean age was (36 ± 16). Most of the male participants had primary education 63%. The majority of females were illiterate 57.1%. Urban residencies were 72.7% (Table 2).

**Table 2:** Socio-demographic characteristics of patients

**Characteristics**	**Male (n = 170)**		**Female (n = 145)**		**Total (n = 315)**	
	**No**	**%**	**No**	**%**	**No**	**%**
Age group						
18-25	42	43.3	55	56.7	97	30.8
26-35	56	57.7	41	42.3	97	30.8
36-45	30	50.0	30	50.0	60	19.0
>45	42	68.9	19	31.1	61	19.4
Mean age	38		33		36	
SD	±16		±14		±16	
Education						
Illiterate	30	42.9	40	57.1	70	22.2
Primary	51	63.0	30	37.0	81	25.7
Secondary	61	51.7	57	48.3	118	37.5
University	28	60.9	18	39.1	46	14.6
Residence						
Urban	108	47.2	121	52.8	229	72.7
Rural	62	72.1	24	27.9	86	27.3

SD = standard deviation.

The socio-demographic characteristics of the medical graduates are summarized in Table 3. A total number of 105 medical graduates participated in the study; among them 61.9% were females. The mean age was 28.8 ± 2.30 years; ranging from 25 to 32 years. The graduates’ experience ranged from three months to four years. The highest proportion of the graduates in this study was those with four years of experience 41.9%, followed by those with 2-3 years of experience 38.1%. The majority of the graduates 58.1% work in public hospitals where 54.30% of them work in the outpatient clinic whereas the remaining 45.70% work in the inpatient. The distribution of the physicians in outpatient and in inpatient depends on work load in the different sites and is liable for change from time to time as perceived by the hospital administration. The majority of the graduates 47.6% work in internal medicine while the remaining work in pediatrics 21%, surgery 21% and obstetrics 10.5%.

**Table 3:** Socio-demographic characteristics of the medical graduates

**Characteristics**	**Male (n = 40)**		**Female (n = 65)**		**Total**	
	**No**	**%**	**No**	**%**	**No**	**%**
**Age group**						
25-27	11	34.4	21	65.6	32	30.5
28-30	13	31.0	29	69.0	42	40.0
>30	16	51.0	15	48.4	31	29.5
Mean age	29 years		28.6 years		28.8 years	
SD	±2.366		±2.114		±2.30	
**Experience (years)**						
<2	6	28.6	15	71.4	21	20.0
2-3	14	35.0	26	65.0	40	38.1
4	20	45.5	24	54.5	44	41.9
**Hospital**						
Public	20	32.8	41	67.2	61	58.1
Private	14	42.4	19	57.6	33	31.4
Both	6	54.4	5	45.5	11	10.5
**Specialty**						
Medicine	23	46.0	27	54.0	50	47.6
Surgery	14	63.6	8	36.4	22	21.0
Pediatric	3	13.6	19	86.4	22	21.0
Gyn/Obs	0	.0	11	10.5	11	10.5
**Location**						
Inpatient	16	40.0	32	49.2	48	45.7
Outpatient	24	60	33	50.8	57	54.3

SD = standard deviation.

Female patients were less likely than males patient to rate the medical graduates’ professionalism and communication skills at a very good 35.2% versus 37.1%, but was not statistically significant. Patients’ age group and education level were strongly associated with their perception of the graduates’ performance. Among the age group of >45 years, 52.5% rated the graduates’ competency as *very good* (p: 0.001), Table 4.

**Table 4:** Patients’ assessment of the medical graduates’ professionalism and communication skills by gender, age, residency and education level

**Variables**	**Patient’s assessment**										
	**Very good**		**Good**		**Inadequate**		**Total**				
	**No**	**%**	**No**	**%**	**No**	**%**	**No**	**%**	**df**	**χ2**	**p**
Gender											
Male	63	37.1	74	43.5	33	19.4	170	54.0	1	.307	.579
Female	51	35.2	77	53.1	17	11.7	145	46.0			
Age group											
18-25	31	32.0	60	61.6	6	6.2	97	30.8	3	30.5	.001*
26-35	36	37.1	36	37.1	25	25.8	97	30.8			
36-45	15	25.0	36	60.0	9	15.0	60	19.0			
>45	32	52.5	19	31.1	10	16.4	61	19.4			
Education											
Illiterate	28	40.0	28	40.0	14	20.0	70	22.2	3	14.0	.003*
Primary	37	45.7	34	42.0	10	12.3	81	25.7			
Secondary	42	35.6	63	53.4	13	11.0	118	37.5			
University	7	15.2	26	56.5	13	28.3	46	14.6			
Residency											
Urban	79	34.5	110	48.0	40	17.5	229	72.7	1	1.78	.182
Rural	35	40.7	41	47.5	10	11.6	86	27.3			

df = (degree of freedom).

(H) =χ2: Kruskal Walli test.

*p statistically significant < 0.05.

The level of patients’ rating the graduates’ skills was inversely associated with their education level. This is illustrated in Table 4, where 40% of the illiterates perceived the graduates communication skills and professionalism as very good. In contrast, only 15.2% of the participants with university education rated them as very good (p: 0.003).

Table 5 shows the mean values of the patients’ assessment of the various items of professionalism and communication skills, which ranges from 3.29 to 3.83. The items dealing with the ability of establishing adequate communication with patients (10, 2 and 3) scored the highest mean values: 3.83, 3.80 and 3.75 respectively: while the items addressing patients’ involvement in decision-making and respect of patient autonomy (9, 8 and 7) scored relatively low mean values 3.54, 3.52 and 3.29 respectively.

**Table 5:** The mean values of patients’ assessment of the medical graduates’ professionalism and communication skills

**How was the physician’s performance at:**	**Assessment**	
	**Mean**	**±SD**
1. Telling you every thing; being truthful, upfront and frank; not keeping things from you that you should know	3.65	1.164
2. Greeting you warmly; calling you by the name you prefer; being friendly, never crabby or rude	3.80	1.158
3. Treating you like you’re on the same level; never “talking down” to you or treating you like a child	3.75	1.111
4. Letting you tell your story ; listening carefully; asking thoughtful questions; not interrupting you while you’re talking	3.69	1.125
5. Showing interest in you as a person; not acting bored or ignoring what you have to say	3.69	1.156
6. Warning you during the physical exam about what he/she is going to do and why; telling you what he/she find	3.70	1.161
7. Discussing options with you; asking your opinion; offering choices and letting help decide what to do; asking what you think before telling you what to do	3.29	1.319
8. Encourage you to ask questions; answering them clearly; never avoiding your questions or lecturing you	3.52	1.189
9. Explaining what you need to know about your problems, how and why they occurred, and what to expect next	3.54	1.181
10. Using words you can understand when explaining any technical medical terms in plain language	3.83	1.136

SD = stander deviation.

## Discussion

The objective of this study was to assess the medical graduates’ professionalism and communication skills from patients’ perspective and to examine its association with patients’ socio-demographic variables. Patients’ opinion as an important dimension of the evaluation of the professional performance and the quality of care has been emphasized by several authors [1-4]. There is increasing evidence that professionalism and communication skills constitute an indispensable quality for any practicing physician. It covers the following requirements: integrity, honesty, compassion, a commitment to keeping current with medical advances, and the ability to communicate effectively with patients, and respect their autonomy [7-12].

The findings of the current study revealed that the mean score value of the *overall* patients’ rating of medical graduates’ skills was 3.64 on five-points Likert scale. Lower mean score values 3 and 3.1 were reported from Kuwait by Bu-Alayyan et al. and Al-Doghaither et al. [40,41] respectively. Much lower mean score value 2.45 was reported from Saudi Arabia [42]. On the other hand higher mean score values 4.86, 4.81 and 3.81 were reported from the USA by Wood et al. [43], Stewart et al. [44] and Moor et al. [18] respectively. This wide variation in the findings might be explained by the different study designs, different instruments for data collection and the different settings in which the studies were conducted.

Several studies indicate that the socio-demographic variables could have significant impact on patients’ expectations, satisfaction and perception of the quality of medical care [45,46]. Our study showed that patients of older age group reported significantly higher level of rating than those of younger age groups. This finding is consistent with what has been reported by Campbell et al. and by Kong et al. [45,46]. The lower level of rating among younger age groups might be explained by the critical attitude and higher expectations of these age groups. Illiterate patients and those with lower educational levels gave significantly higher scores of rating than those with higher educational levels. This finding is supported by a similar study conducted by Fiscella [47]. The difference in rating by educational level could be explained by the greater exigencies of the highly educated patients.

Establishing adequate communication with the patient has been identified as a corner-stone in the doctor patient relationship, and constitutes a pre-requisite for appropriate diagnosis, management and quality care, which in turn positively impacts patients’ satisfaction [16,48]. Also, patients’ involvement in decision-making is widely recognized as an expression of patient respect and patient autonomy [49-51]. In the current study, patients rated the different items of graduates’ professionalism and communication skills at a good level. *The items assessing the ability of establishing adequate communication with patients* scored the highest mean values, which is similar to what has been reported by Clever et al. [52]. On the other hand a relatively lower mean score values were given to *patient involvement in decision–making.* Sekimoto et al. [53] from Japan reported higher mean score values in this area of competency. The findings of our study could be explained by the dominance of the paternalistic approach in the doctor patient relationship in the clinical practice environment in which the graduates perform their duties. This interpretation is consistent with what has been reported by Roter et al. and Braddock et al. [54,55].

Further studies are recommended for better understanding of the factors that influence patients’ perception of the quality of communication skills and professionalism of the medical graduates.

### Study limitations

Due to constraints of time and resources, this study covered only the graduates working in Aden hospitals. However, the medical graduates of Aden university are deployed allover the country, both in urban and rural areas that have wide variation in working conditions, and facilities. To have more reliable assessment, it is recommended that future studies include larger and more representative samples that cover different areas and settings of practice. Also, this study did not investigate the organizational and structural features of outpatients’ clinics and hospital wards which might have significant influence on physicians’ performance and the overall quality of care and consequently on patients’ rating level of professionalism and communication skills [43-45]. Therefore, these aspects need to be addressed in future studies.

## Conclusion

Generally, patients rated the graduates’ professionalism and communication skills at a good level. Patients’ age and educational level were positively associated with the rating level. Younger age groups and those with higher education were more critical in their assessment. The highest rating scores were observed for the items assessing the ability of establishing adequate communication with patient and the minimum scores for those dealing with patient involvement in decision-making. The findings of this study are very important for curriculum reforms that should address the identified areas of competency that need further improvement as well as for continuing professional development programs. Graduates-assessment should be conducted on regular basis, with feedback for all stakeholders in order to make the necessary interventions for promoting the professional competency and the quality of care. Further studies on larger samples and different settings are recommended.

## Competing interest

The authors declare that they have no competing interests.

## Authors’ contributions

FTA was involved in the conception, design, analysis and interpretation of data, report writing and manuscript writing. ASH was involved in the conception, design and manuscript review. Both authors have read and approved the final version of the manuscript.

## Pre-publication history

The pre-publication history for this paper can be accessed here:

http://www.biomedcentral.com/1472-6920/14/28/prepub

## Supplementary Material

Additional file 1 American Board of Internal Medicine Using Patients Assessment for Continuous Professional Development. Click here for file
