# Supplementary material for: Patients’ assessment of professionalism and communication skills of medical graduates
Source: BMC Med Educ. 2014 Feb 11;14:28. doi: 10.1186/1472-6920-14-28 (PMC3923249; doi:10.1186/1472-6920-14-28)
Supplement: Additional file 1 — American Board of Internal Medicine Using Patients Assessment for Continuous Professional Development. [file 1472-6920-14-28-S1.doc]

**Additional file 1:**
